# High-Throughput Sequencing Supports Strong Geographical Patterns in the *Cladia aggregata* Complex (Ascomycota, Lecanorales) and Identifies the Asian Clade as an Independent Species

**DOI:** 10.3390/jof12020090

**Published:** 2026-01-28

**Authors:** John de Abreu, Alejandrina Barcenas-Peña, Vasun Poengsungnoen, Xinyu Wang, Jen-Pan Huang, Helge Thorsten Lumbsch, Felix Grewe

**Affiliations:** 1 College of Science and Health, DePaul University, Chicago, IL 60614, USA; 2 The Grainger Bioinformatics Center & Negaunee Integrative Research Center, Science & Education, The Field Museum, Chicago, IL 60605, USA; abarcenas@fieldmuseum.org (A.B.-P.); tlumbsch@fieldmuseum.org (H.T.L.); 3 Department of Biology, Faculty of Science, Ramkhamhaeng University, Bangkapi, Bangkok 10240, Thailand; vasunpoeng@gmail.com; 4 State Key Laboratory of Phytochemistry and Natural Medicines, Kunming Institute of Botany, Chinese Academy of Sciences, Kunming 650201, China; wangxinyu@mail.kib.ac.cn; 5 Biodiversity Research Center, Academia Sinica, 128 Academia Rd., Section 2, Nankang District, Taipei 11529, Taiwan; jhuang@fieldmuseum.org

**Keywords:** next-generation sequencing, systematics, phylogenetics, molecular variation, biogeography

## Abstract

The *Cladia aggregata* group of lichen-forming fungi comprises multiple species that are difficult to differentiate based on phenotypic characters. It has a wide distribution across several continents, but is most diverse in Australasia. We aimed to delimit the species complex further, investigate the relatedness of the lineages, and examine their distributional ranges and phenotypic traits. We used Restriction Site Associated DNA Sequencing (RAD-seq) to compare thousands of loci across 91 individuals from the Americas, Asia, and Australasia. All Asian samples formed a distinct, monophyletic clade in all phylogenetic trees, while the American samples divided into two clades, one comprising South American samples and another comprising Caribbean samples, with the latter representing *C. aggregata* sensu stricto, as the type specimen was collected in Jamaica. Further population-genomic analyses support the conclusion that the Asian samples are genetically distinct and are here described as a new species. The new species, *Cladia asiatica,* accommodates the Asian samples previously included in *C. aggregata*. Our analysis highlights the potential of next-generation sequencing to reveal hidden diversity and resolve the phylogeny of this species complex and lichen-forming fungi in general.

## 1. Introduction

Taxonomy has three primary roles in science: assigning organisms to recognized species, organizing species into higher categories such as genus and family, and delimiting species. Species delimitation is the designation of species when individuals do not adhere to current species categories. Accurate species delimitations are necessary for ecological and conservation research [1]. However, estimates suggest that a substantial proportion of the world’s species remain hidden, with potentially 86% of terrestrial species and 91% of marine species yet to be described [2].

Among these yet-undescribed organisms are many lichens, which are stable symbiotic associations between a fungal partner (the mycobiont) and a photosynthetic partner (the photobiont) [3]. Traditionally, species delimitation of lichen-forming fungi has relied on morphological and chemical traits. While this approach is helpful for some taxa, it can be challenging for those with few distinctive traits or high phenotypic plasticity [1]. Many cryptic species of lichen-forming fungi likely remain hidden within phenotype-based species descriptions, reflecting substantial unexplored or undiscovered diversity [4].

Next-generation sequencing has successfully delimited species complexes that were previously difficult to distinguish through multi-locus, morphological, or chemical approaches. This technology allows for the in-depth exploration of numerous single-nucleotide polymorphisms (SNPs) across the entire genome [5]. In particular, restriction site-associated DNA sequencing (RAD-seq) has improved our understanding of the number of lichen-forming fungus species, their phylogenetic relationships, and their geographic distribution [6,7,8].

The *Cladia aggregata* group includes fruticose lichens belonging to the family *Cladoniaceae* [9] that exhibit substantial morphological variation. Different taxonomic approaches have been used to interpret or classify this variation. While Filson [10] applied a broad species concept, subsequent authors separated certain taxa from *C. aggregata* s. lat. based on morphological and chemical traits [11,12]. The type specimen was collected in Jamaica in 1788 by the Swedish botanist Olof Peter Swartz [13]. The species has a wide distribution, primarily in the Southern Hemisphere [14].

Recently, the *C. aggregata* species complex was studied using multi-gene analyses, leading to the formal description of additional species within the complex [15,16]. Currently, 12 species are recognized in the group, but distinct geographic patterns have been found in the remaining clades within *C. aggregata* sensu stricto [15]. We here revisited this *C. aggregata* group, with special emphasis on Asian populations and their relationships to Neotropical populations from which the type material is derived. We employed RAD-seq data, which have been successfully used to disentangle phylogenetic relationships in taxonomically difficult groups, such as the neuropogonoid species in *Usnea* [7,8] and species pairs in the genus *Usnea* [17].

We addressed the following research questions by using RAD-seq on the *C. aggregata* species complex: (1) Are there more species within the complex than previously identified? (2) How are these species related to previously described species? (3) What is the geographic range of these species? (4) Which phenotypic traits correspond to genetic variation within the complex? We hypothesized that the Asian and Australian populations represent a separate species from the neotropical populations. The wide geographic range of the species complex suggests that gene flow between geographically separated populations may be limited.

## 2. Materials and Methods

### 2.1. Taxon Sampling

A total of 95 specimens of *Cladia aggregata* were sequenced for this study, representing its broad geographic distribution across the Neotropics, Australasia, East Asia, and Thailand (Table S3).

As an initial outgroup, we included specimens of *Pulchrocladia ferdinandii* and *P. retipora*. However, based on preliminary phylogenetic results and a morphological reassessment, these samples were removed and replaced with three specimens identified as *Cladia neocaledonica* (C1011219F, C2035641F, and C2035148F), which provided a more appropriate, closely related outgroup within the same genus.

Following sequencing and data processing, one sample (C1011230F) was excluded due to low data coverage (<200 loci), and another sample (RAMK40318) was removed because of a high estimated sequencing error rate (0.00648). The final dataset, therefore, comprised 91 samples.

Of these, five samples had been included in previous studies of the *C. aggregata* complex [16] and were previously identified as *C. inflata*, *C. blanchonii*, *C. terebrata*, or *C. neocaledonica* (Table S1). Subsequent morphological examination confirmed the identification of two additional specimens from Argentina and New Zealand as *C. inflata* (Table S1).

### 2.2. NGS Sequencing and Initial Data Processing

DNA extractions and the construction of the RAD-seq library were performed at the University of Wisconsin-Madison Biotechnology Center. DNA was extracted using the DNeasy mericon Food Kit, and the RAD-seq library was prepared using the restriction enzyme ApeKI before sequencing on an Illumina Novaseq 6000 (Illumina, San Diego, CA, USA) following a procedure similar to [18]. The RAD-seq data were received as gzipped fastq files.

The RAD-seq data were processed using ipyrad v0.9.90 using the servers (Dell Inc., Round Rock, TX, USA) of the Grainger Bioinformatics Center at the Field Museum as previously described in Otero et al. (2023) [8]. We set the ipyrad parameter files for paired GBS data, the assembly method to reference, and the ploidy to 1 (haploid). We kept the minimum sample coverage of 4. The reference sequence was the genome of *Cladonia squamosa* from the Wellcome Sanger Institute, which was downloaded from GenBank (GCA_947623385.1). The reference sequence was used to filter reads from the lichen-forming fungus from the rest of the metagenome. Sequences that could not be mapped to the reference genome were discarded by ipyrad.

### 2.3. Phylogenetics

RAxML (Randomized Axelerated Maximum Likelihood) is a maximum likelihood method for reconstructing phylogenetic trees. We used RAxML v8.2.12 [19]. We initially made a tree with 95 samples prior to removing two samples for poor data quality and the two outgroup samples, because three samples later identified as *C. neocaledonia* functioned as a better outgroup. The input data was the phylip file of 91 samples created by ipyrad. The phylip or .phy file contains all the loci from the data concatenated into a supermatrix with missing data filled in as Ns [20]. 1000 bootstraps were run for each analysis using the GTRCATX substitution model. See Supplementary Materials Figure S2 for the RAxML tree. Additionally, we used IQ-TREE v2 [21] as another method to calculate maximum-likelihood trees using the same input file. We used the model checker [22], which assigns BIC values to multiple different nucleotide substitution models to choose the best model for the specific data entered. The ultrafast bootstrap [23] was used to reduce the computation time. We used the best-fit model based on BIC values (TPM3u + F + R5) and ran 1000 bootstraps. The output trees from both maximum-likelihood methods were visualized using FigTree v1.4.4 [24]. The trees were rooted at the midpoint, then the node order was set to “increasing” for both trees.

SVDquartets [25] is a coalescent-based method implemented in the PAUP v4.0a package [26]. SVDquartets calculates several quartet trees, each using 4 SNPs, before using those trees to make an overall species tree. We used PAUP to convert the file of unlinked SNPs to a Nexus format for our input file. We used CASTER-site v1.20.2.5 (Coalescence-aware Alignment-based Species Tree EstimatoR) [27] to assign bootstrap values to the SVD Quartets tree. In CASTER-site, we used the newick-formatted output tree from SVDquartets as a constraint tree and the phylip file from ipyrad as a loci matrix. Due to the speed of CASTER-site, we set the argument for the number of placements (-r) and the number of rounds of subsampling (-s) to 10,000. We used FigTree [24] to visualize the results. The branches were converted to a cladogram, the node order set to “increasing”, and the tree was manually rooted with the outgroup. We used different software, each using different models to calculate maximum likelihood trees and a coalescent-based method to calculate another tree, so that we had multiple lines of evidence to further support our conclusions of the relationships between *C. aggregata* complex populations.

### 2.4. Statistics

Statistical analyses were conducted on a reduced dataset only comprising samples from the American and Asian clades. Other samples were excluded because they formed small clades, showed inconsistent topologies across phylogenetic methods, or had low bootstrap support. We used the R package poppr v2.9.7 [28] to run an AMOVA (Analysis of Molecular Variance). The AMOVA was used to calculate the genomic variation within and between clades. We also used the ade4 package v1.7.23 [29] to compare the variation between our assigned groups and randomly assigned groups. We used the randtest command for this comparison, which uses a Monte Carlo test to simulate randomly assigned groups of samples and ran 999 iterations. Our input file was a genind object generated with the adegenet v2.1.11 package [30]. The genind object was made from a vcf file recoded using VCFtools v0.1.16 [31] so that sites found in less than 50% of the data and those with a minor allele frequency below 5% were excluded.

### 2.5. Population Genetics

All population-genomic analyses were performed using the same reduced dataset described above. A Principal Component Analysis (PCA) was performed to summarize genetic variation between samples using the same genind object as used before. We used the dudi.pca function in ade4 [29] to calculate the PCA. values were replaced with the mean allele frequencies. We used ggplot2 v3.5.2 [32] to visualize the plot.

We used fineRADstructure v0.3.2 to group samples into estimated populations based on co-ancestry or relatedness among individuals. FineRADstructure can show shared ancestry in more detail than similar methods, including the level of shared ancestry between populations rather than just the number of separate populations [33]. The ipyrad alleles file was converted to a fineRADstructure file using the finerad_input.py script in the fineRADstructure tools. The minimum number of samples per locus was set to four. RADpainter was used to calculate a co-ancestry matrix for the data. We used fineSTRUCTURE to group individuals into populations using an MCMC clustering algorithm. The number of sample iterations (-y) was set to 100,000, the number of burn-in iterations (-x) was set to 100,000, and the thin interval (-z) was set to 1000. The method (-m) was set to the default, which performs the full range of moves to obtain the best posterior state. To visualize the tree with estimated population sizes, we used the R packages ape v4.4.3 [34] and XML v3.99.0.18 [35].

FastSTRUCTURE v1.0 [36] was used to measure admixture between populations. The dataset was initially cleaned with VCFtools v0.1.16 [31] as described in the Statistics section. We used PLINK v1.9 [37] to convert the newly created vcf files into plink files. The sample assignment was studied across different population sizes (K values). Then we ran fastSTRUCTURE for K = 1–10. The R package POPHELPER v2.3.1 [38] was used to visualize results grouped by population and to view Fast Structure results for several K values simultaneously.

### 2.6. Phenotypical Characteristics

We performed high-performance thin-layer chromatography (HPTLC) on all samples. All 95 samples, including the outgroup, were soaked in acetone and chromatographed using solvent B and C [39]. We used the traits described in [11] to measure morphological differences among the samples in this study. These traits include the shape of apices, perforation of samples, sample color, branch pattern, and others. We followed a study that assigned a numerical value to each possible option for every trait, for example, samples with abundant perforations are assigned 1, and those with little to no perforations are assigned 0 for that trait [15]. Assigning numerical values to each trait enabled us to create a matrix of morphological traits, allowing us to quantify the physical differences between samples.

### 2.7. Biogeography

RASP v4.4 [40] is a software that can apply several methods of estimating the geographic distribution of ancestral nodes in phylogenies. The treefile from IQ-TREE 2 was provided as a consensus tree. RASP requires a states file, and we provided a text file in the format recommended for BioGeoBears [41]. We limited the number of states a node could occupy to two areas for our analysis. We ran BBM (Bayesian Binary MCMC) on the consensus tree [42,43]. The number of cycles was 50,000 with 10 chains. The frequencies of samples and discard samples were both set to 100, and the temperature was set to 0.1. The model for the state frequencies was a fixed Jukes-Cantor model, and a gamma distribution with a shape parameter from 0.001 to 100 was selected for among-site rate variation.

## 3. Results

### 3.1. RAD Sequencing

We recovered 5432 loci from the complete set of 91 samples after filtering, yielding an average of 1483.51 loci per sample (Table 1). The average number of raw reads per sample was 7,120,563, while the average number of reads mapped to the reference genome per sample was 187,517.5 (2.6%). After removing four samples, the matrix generated by ipyrad contained 30,841 SNPs, with 79% missing sites. The average error estimate per sample was 0.0017.

### 3.2. Phylogenetic Analyses

**Figure 1:**
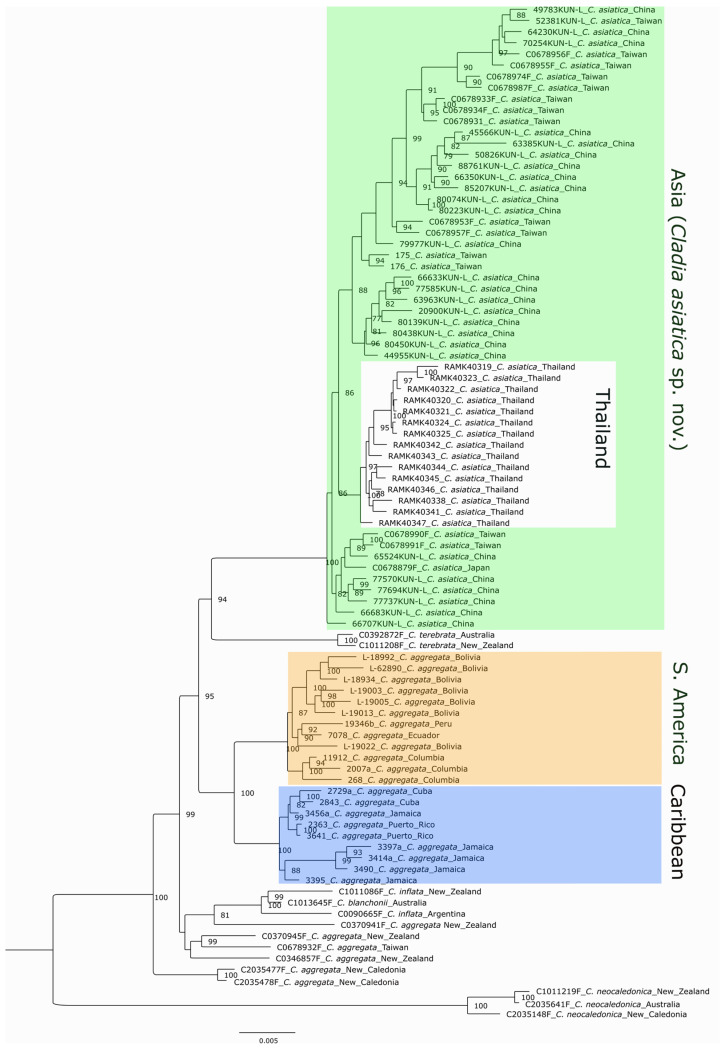
A phylogenetic Maximum Likelihood tree calculated with IQ-TREE v2. Each sample ID is represented in the following format: the accession number, if available, or collection number, followed by the species name and the country where the sample was collected. Bootstrap values above 75 are shown next to their respective branches. The Asian clade corresponds to the here-described *Cladia asiatica* sp. nov.

The maximum-likelihood phylogenetic tree inferred using IQ-TREE v2 is shown in Figure 1. This analysis recovered three major geographically structured clades corresponding to the Caribbean, South American, and Asian regions, each strongly supported. Within the Asian clade, samples from Thailand formed a moderately supported subclade (bootstrap = 86). The remaining Asian samples from China, Taiwan, and Japan were divided into two weakly supported and paraphyletic lineages. A Chinese specimen 66707 KUN-L was recovered as a sister to all other Asian samples.

Two taxa identified as *C. terebrata* from Australia and New Zealand formed a well-supported sister group to the Asian clade. Together, the Asian, South American, Caribbean, and *C. terebrata* lineages were recovered as sister to a clade composed predominantly of Australasian taxa, together with one *C. inflata* sample from southern Argentina and one sample from Taiwan. This latter group was not supported as monophyletic in the RAxML or coalescent-based analysis. A New Caledonian clade was resolved as the earliest-branching lineage in the tree and received strong bootstrap support.

The RAxML maximum-likelihood analysis (Figure S1) recovered an overall topology largely congruent with the IQ-TREE reconstruction, including strong support for the same three major geographically structured clades (Caribbean, South American, and Asian), and likewise resolved the New Caledonian clade as the earliest-branching lineage. However, several differences were observed in the internal structure of the Asian clade and in the relationships among Australasian taxa.

In contrast to the IQ-TREE reconstruction, Thai samples formed a sister group to an unsupported clade comprising all other Asian samples, although the Thai subclade itself remained strongly supported (bootstrap = 100). In addition, the Chinese specimen 66707 KUN-L was placed within the Asian clade rather than as sister to the remaining Asian samples. The arrangement of Australasian taxa also differed between the two analyses. Whereas these samples formed an unsupported clade in the IQ-TREE reconstruction, they were recovered as paraphyletic in the RAxML tree. In the latter analysis, two samples identified as *C. inflata* and one sample of *C. blanchonii* formed a moderately supported sister clade (bootstrap = 85) to all remaining taxa.

The coalescent-based tree (Figure S2) also shows strong support for the major clades recovered in the Maximum Likelihood analyses (Figure 1 and Figure S1), including the Asian, South American, Caribbean, *C. terebrata*, and New Caledonian clades. However, it differed in several key aspects, particularly in the placement of basal lineages: the Thailand and East Asian subclades within the Asian lineage received strong support, whereas their relationships were only weakly and inconsistently resolved in the ML trees (Figure 1 and Figure S1). The *C. terebrata* clade was recovered as the earliest-branching lineage, sister to all other taxa. One *C. inflata* sample clustered with the *C. blanchonii* specimen as sister to the Asian clade. This placement corresponds to the position occupied by the *C. terebrata* clade in the Maximum Likelihood analyses (Figure 1 and Figure S1). The remaining *C. inflata* sample grouped with the Australasian and New Caledonian taxa, which together formed a paraphyletic assemblage.

### 3.3. Sampling for Population Genomics Analyses

Samples for population genomics analyses were selected based on the large, well-supported clades identified in our maximum-likelihood and coalescent-based phylogenies (Figure 1, Figures S1 and S2): Thailand, East Asia, South America, and the Caribbean. These four groups contained 77 samples, and the filtered dataset included 1542 loci and 3084 alleles.

### 3.4. Population Genetics

Population genomic analyses of the reduced dataset revealed strong genetic structure among samples from Thailand, East Asia, South America, and the Caribbean. AMOVA showed that most genetic variation (79.19%) was attributable to differences among geographic groups, whereas only 20.81% of the variation occurred within groups (Table 2). This pattern was supported by a Monte Carlo test, which indicated that the observed variance among the predefined groups was substantially higher than the variance expected under random grouping. The observed among-group variance (544.98) was substantially greater than the average variance of simulated groups (170.36), and the test was highly significant (*p* = 0.001; Table 2). These results confirm pronounced population differentiation corresponding to the major phylogenetic clades.

**Figure 2:**
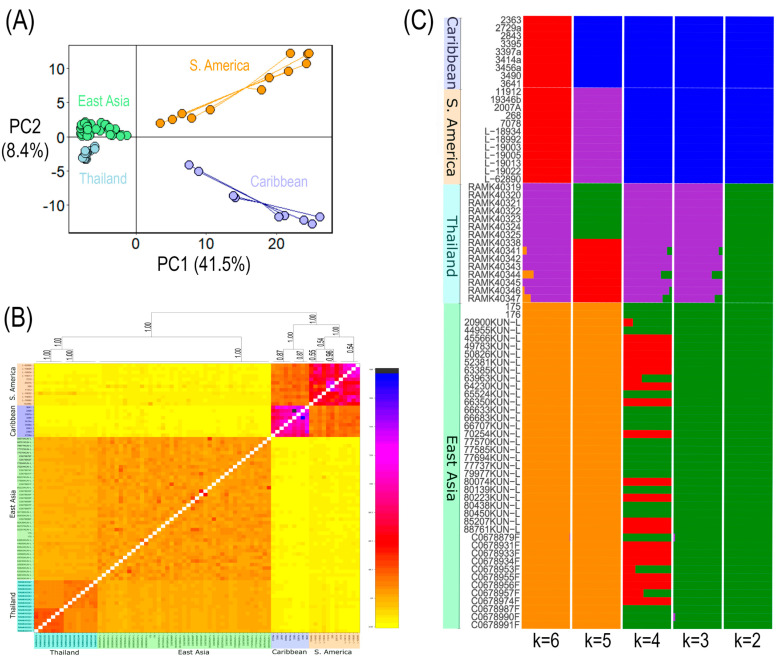
Population structure of *Cladia aggregata* samples from Asia and the Americas. (**A**) Principal component analysis (PCA) of Asian and American samples, with PC1 (*x*-axis) explaining 41.5% and PC2 (*y*-axis) explaining 8.4% of the total genetic variation. (**B**) Co-ancestry heatmap generated with fineRADstructure, showing relatedness among individuals. The dendrogram above the plot represents the hierarchical clustering of samples inferred by fineRADstructure. (**C**) fastSTRUCTURE results for K = 2–6. Each horizontal bar represents an individual sample, with colors indicating inferred genomic ancestry components. Results are grouped by K value (*y*-axis) and population (*x*-axis).

The PCA supports the separation of samples from Asia and the Americas. PC1 explains 41.5% of the total genetic variation and clearly separates Asian from American samples (Figure 2A). Asian samples form a more compact cluster, whereas samples from the Americas are more broadly dispersed along this axis. PC2 explains 8.4% of the variation and further differentiates East Asian and Thai samples, as well as South American and Caribbean samples, although this separation is less pronounced (Figure 2A).

Using fineRADstructure (Figure 2B), we assessed patterns of co-ancestry among individuals to evaluate genetic structure within and among major clades. The resulting co-ancestry matrix resolved distinct clusters corresponding to Thailand, East Asia, South America, and the Caribbean. Samples within each of these four groups showed higher relatedness to one another than to samples from other groups. The Caribbean and South American groups exhibited stronger within-group co-ancestry than the East Asian and Thai groups. Co-ancestry between the Asian and American samples was low, indicating limited recent gene flow between regions. Although a separation between East Asia and Thailand, and between South America and the Caribbean, was detected, these divisions were comparatively weak.

Population patterns and gene flow indicated by co-ancestry were also observed with fastSRUCTURE v1.0 (Figure 2C). As with other population-genomics analyses, fastSTRUCTURE results clearly separated samples from Asia and the Americas. K = 3 through 6 also separated Thailand from East Asia, although only K = 5 showed no admixture between East Asia and Thailand, while K = 5 also separated the Caribbean and South America.

### 3.5. Phenotypic Characteristics

The *C. aggregata* complex exhibits extensive intraspecific morphological variation, much of which is non-diagnostic and therefore unsuitable for reliable species delimitation.

Chemical characters likewise did not provide additional resolution among species. Most samples produced the same secondary metabolites and belonged to chemotype 1. Samples producing compounds distinct from chemotype 1 were largely identified as belonging to species other than *C. aggregata* sensu stricto. The few samples still assigned to *C. aggregata* sensu stricto that exhibited alternative chemotypes originated from a single locality in Bolivia. Consequently, chemical variation did not correspond to species boundaries or geographic structure in this dataset. Details of chemotype composition and geographic distribution are provided in Supplementary Table S2.

### 3.6. Biogeography

**Figure 3:**
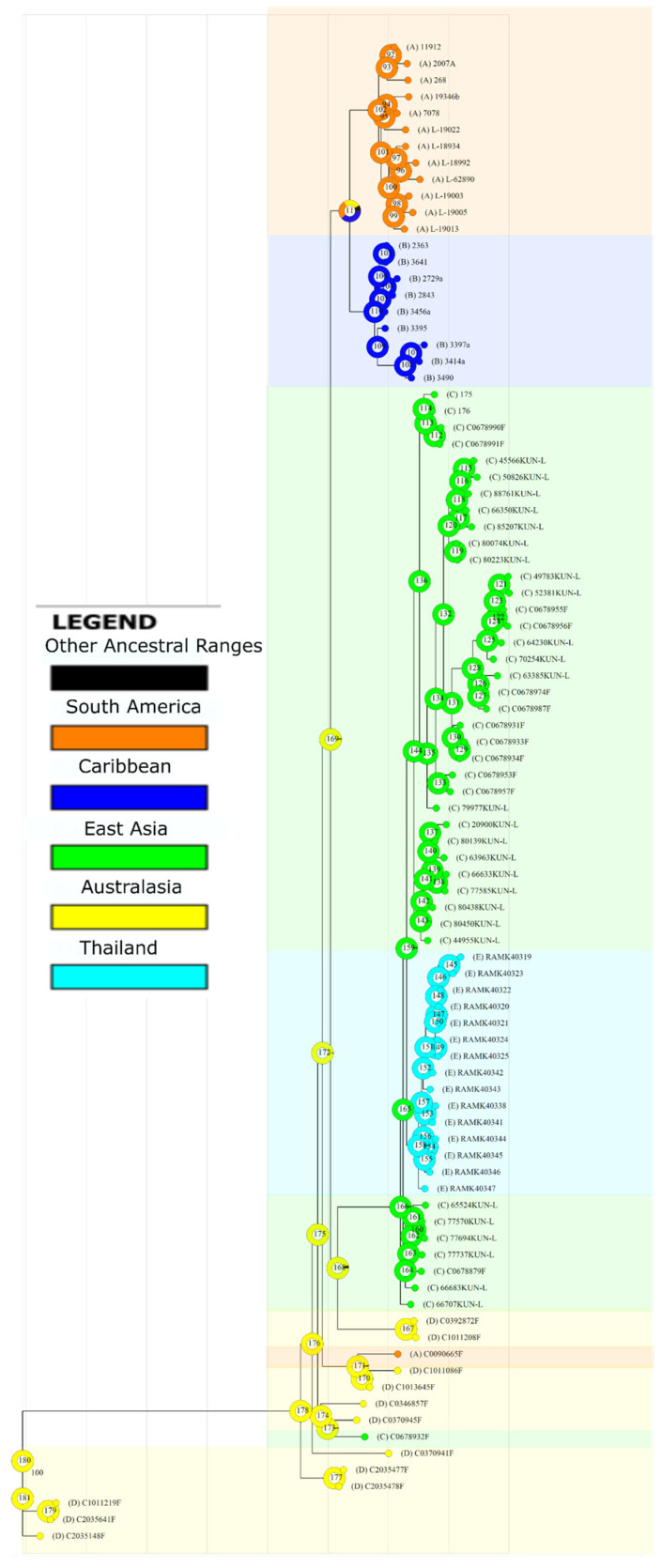
Ancestral area reconstruction inferred using Bayesian Binary MCMC (BBM) implemented in RASP, based on the Maximum Likelihood consensus tree obtained with IQ-TREE v2. Pie charts at internal nodes indicate posterior probabilities of ancestral geographic ranges. Colors represent the predefined geographic regions: South America (orange), Caribbean (blue), East Asia (green), Australasia (yellow), and Thailand (cyan). Black portions of pie charts correspond to alternative ancestral range combinations with posterior probabilities below the display threshold. Terminal taxa are colored according to their present-day geographic origin.

Range reconstruction suggests that the ancestral populations of *C. aggregata* originated in Australasia (Figure 3). The BBM results indicate that dispersal to Asia and the Americas was more recent compared to ancestral splits within Australasian populations. BBM also estimates that the common ancestor of both the Thai and East Asian populations was most likely from East Asia. However, the common ancestor of the American populations remains unclear and may have originated in South America, the Caribbean, or Australasia, before dispersing independently to both regions.

### 3.7. Taxonomy

**Figure 4:**
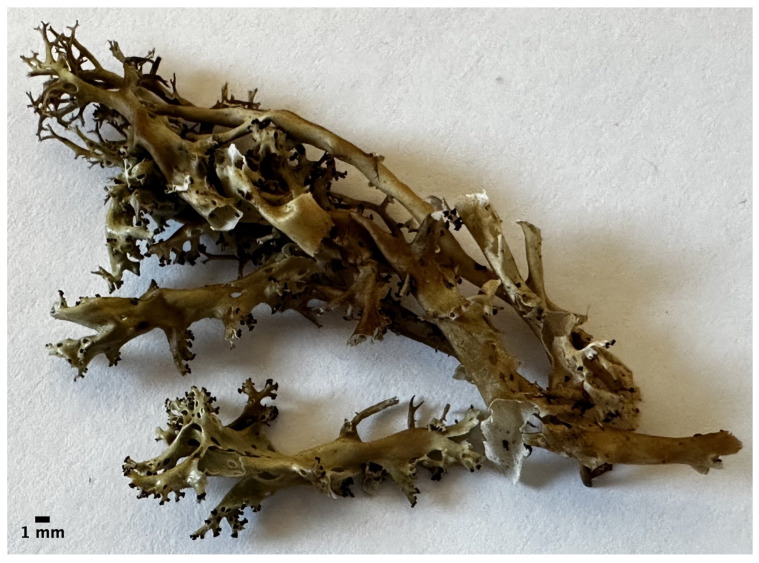
Habit of *Cladia asiatica* sp. nov. (Holotype). This is sample RAMK 40342 an example of the overall appearance of the proposed species *C. asiatica*.

*Cladia asiatica* de Abreu, Barcenas-Peña, Poengsungnoen, Wang, Huang, Lumbsch & Grewe (Figure 4).

MycoBank: MB861532.

Diagnosis: Thallus fruticose with hollow, glossy pseudopodetia in loose clusters, pale yellow-green to brownish. Sterile pseudopodetia slender and mostly dichotomously branched; fertile pseudopodetia robust, inflated, racemosely branched, with abundant perforations. Apothecia and pycnidia frequent. Contains barbatic acid and 4-*O*-demethylbarbatic acid. Differs from the morphologically similar *C. aggregata* s.str. by phylogenetic data. Restricted to East Asia.

Type: THAILAND. Loei Province, Phu Luang Wildlife Sanctuary, Khok Nok Kraba Ranger Station, Tha Sala, Phu Ruea, 17°16′39.4″ N, 101°31′33.3″ E, 1555 m, lower montane scrub forest, on moss, 26 June 2008, K. Buaruang KBPL005 (RAMK 40342) (holotype—RAMK, isotype—F); K. Buaruang KBPL001 (RAMK 40338), KBPL007 (RAMK 40343), W. Polyiam WPPL006 (RAMK 40344) (paratypes—RAMK).

Etymology: The epithet refers to the geographical range of the new species, which occurs in Asia.

Description: Thallus fruticose, consisting of hollow, loosely clustered pseudopodetia, forming clumps, tufts or swards; glossy, pale yellow to green or greenish brown or blackish brown; surface smooth, rarely somewhat dimpled, becoming striate in older thalli. Sterile pseudopodetia, variable, erect, ascending or decumbent, slightly inflated and tapered, (3–)10–80(–110) mm tall, (0.1–)0.5–1.5(–5) mm wide; branching, dichotomous toward the apex; perforations sparse to numerous, scattered or to one side of the pseudopodetia, slit-like to roundish or oval, (0.1–)0.5–2 mm wide; medullary cavity white, farinose. Fertile pseudopodetia erect to decumbent, inflated, typically more robust and taller than sterile pseudopodetia, 12–80 mm tall, 1.5–3.5(–5) mm wide; branching, racemose or corymbose; perforations rare to abundant, 0.2–1.5 mm wide. Apothecia apical, 0.1–0.2 mm wide, clustered in groups of up to c. 6–12. Ascospores ellipsoid, (6–)7–11 (–12) × 2.5–4μm. Pycnidia common, at the apices of sterile pseudopodetia or on the lower laterals of fertile pseudopodetia. Conidia filiform to narrowly fusiform, straight or rod-shaped, 5–8 × 0.6–1.5 μm.

Chemistry: Contains barbatic acid and 4-*O*-demethylbarbatic acid.

Distribution: East Asia: China, Taiwan, Thailand, and Japan.

Notes: The new species is morphologically cryptic and difficult to distinguish from *C. aggregata*. However, in the phylogenetic analysis (Figure 1), it forms a strongly supported clade restricted to Asia, which, together with genetic differentiation, provides the basis for its segregation; genetic data are therefore required for reliable identification.

Additional specimens examined: CHINA. Fujian Province, Wuyishan Ci., Wuyishan National Reserve, Huanggangshan Mt., 27°42′30.76″ N, 117°45′02.24″ E, 2160 m, on soil, 26 May 2015, LS Wang et al., 15-46896 (KUN-L 49783). Guangdong Province, Maoming Ci., Dacheng Town, 22°17′21.94″ N, 111°12′57.09″ E, 1626 m, on soil, 28 March 2021, XY Wang et al., 21-69528 (KUN-L 77570). Guangdong Province, Maoming Ci., Dawuling Reserve, 22°18′06.30″ N, 111°13′21.69″ E, 1316 m, on rock, 29 March 2021, XY Wang et al., 21-69652 (KUN-L 77694). Guangdong Province, Maoming Ci., Qianpai Town, 22°17′27.71″ N, 111°13′04.51″ E, 1558 m, on soil, 28 March 2021, XY Wang et al., 21-69543 (KUN-L 77585). Guangdong Province, Shaoguan Ci., Mangshan Forest Park, 24°53′58.39″ N, 113°01′20.52″ E, 1460 m, on rock, 18 May 2019, LS Wang et al., 19-63233 (KUN-L 66707). Guangdong Province, Shaoguan Ci., Nanling National Forest Park, 24°54′58.00″ N, 113°02′22.16″ E, 865 m, on soil, 17 May 2019, LS Wang et al., 19-63159 (KUN-L 66633). Guangdong Province, Shaoguan Ci., Nanling National Forest Park, 24°56′10.19″ N, 113°00′18.43″ E, 1182 m, soil over rock, 17 May 2019, LS Wang et al., 19-63209 (KUN-L 66683). Guangdong Province, Zhaoqing Ci., Dinghushan Reserve, 23°10′31.97″ N, 112°31′11.64″ E, 630 m, on sandy rock, 31 March 2021, LS Wang et al., 21-69695 (KUN-L 77737). Sichuan Province, Daocheng Co., 29°22′01.70″ N, 100°08′09.75″ E, 4395 m, on soil, 12 July 2022, XY Wang & M Ai XY22-1389 (KUN-L 85207). Sichuan Province, Yanyuan Co., along the road from Yanyuan to Xichang, 27°32′11.73″ N, 101°43′34.53″ E, 3135 m, on soil, 10 April 2019, LS Wang et al., 19-62809 (KUN-L 66350). Taiwan Province, Jiayi Ci., Alishan Forest Park, 23°30.653′ N, 120°48.984′ E, 2307 m, on decaying bark, 26 September 2015, LS Wang & XY Wang 15-49383 (KUN-L 52381). Xizang Province, Dingjie Co., Chentang Town, 27°53′53.13″ N, 87°27′25.37″ E, 2930 m, on rock, 28 July 2019, LS Wang et al., 19-65746 (KUN-L 70254). Xizang Province, Motuo Co., Gelin Village, 29°13′20.93″ N, 95°11′12.04″ E, 1555 m, on soil, 24 November 2018, XY Wang & AC Yin 18-61989 (KUN-L 65524). Yunnan Province, Chuxiong Yi Autonomous Prefecture, Wuding Co., 25°37′20.13″ N, 102°12′39.86″ E, 1972 m, on soil, 6 September 2021, XY Wang et al., XY21-11 (KUN-L 80438). Yunnan Province, Chuxiong Yi Autonomous Prefecture, Wuding Co., 25°37′21.21″ N, 102°12′49.09″ E, 1973 m, on soil, 6 September 2021, XY Wang et al., XY21-23 (KUN-L 80450). Yunnan Province, Dali Ci., Ma’er Mt., 26°15′21.67″ N, 100°06′32.21″ E, 3502 m, on soil, 21 June 2018, LS Wang et al., 18-59051 (KUN-L 63385). Yunnan Province, Dali Ci., Shaxi Vil., Shibao Mt., 26°21′31″ N, 99°50′22″ E, 2470 m, on soil, 29 September 2018, LS Wang et al., 18-60455 (KUN-L 63963). Yunnan Province, Fengqing Co., Yaojie Town, 24°34′56.76″ N, 100°06′47.67″ E, 2261 m, on soil, 8 November 2013, D Liu 13-40004 (KUN-L 20900). Yunnan Province, Kunming City, Tangdan Town, Lanniping, 26°10′02.58″ N, 102°57′45.56″ E, 3360 m, on rock, 14 May 2014, LS Wang et al., 14-43698 (KUN-L 45566). Yunnan Province, Lijiang Ci., Laojun Mt., 26°38′30″ N, 99°43′47″ E, 3855 m, on soil, 28 September 2018, LS Wang et al., 18-60722 (KUN-L 64230). Yunnan Province, Luquan Co., along the road from Luquan to Yunlong Reservoir, 25°42′29.66″ N, 102°29′28.08″ E, 1952 m, on rock, 18 April 2014, LS Wang et al., 14-43149 (KUN-L 44955). Yunnan Province, Nanjian Co., Yongcui Vil., 24°53′12.51″ N, 100°21′40.77″ E, 2458 m, on soil, 3 July 2015, X Ye & WC Wang 15-47927 (KUN-L 50826). Yunnan Province, Pu’er Ci., Jingdong Co., 24°32′40.99″ N, 101°01′40.24″ E, 2437 m, on soil, 28 October 2021, XY Wang & M Ai XY21-689 (KUN-L 80223). Yunnan Province, Pu’er Ci., Jingdong Co., 24°32′46.06″ N, 101°01′39.44″ E, 2484 m, on soil, 28 October 2021, LS Wang et al., 21-70633 (KUN-L 80074). Yunnan Province, Xinping Co., Mopanshan Mt., 24°00′17.01″ N, 101°57′03.68″ E, 2181 m, on rock, 25 October 2021, XY Wang & M Ai XY21-605 (KUN-L 80139). Yunnan Province, Xinping Co., Mopanshan Mt., 23°57′39.22″ N, 101°56′14.74″ E, 2035 m, on fallen twigs, 1 May 2023, XY Wang & SY Wang XY23-154 (KUN-L 88761). Yunnan Province, Xinping Co., Mopanshan Mt., 23°57′14.27″ N, 101°57′01.94″ E, 2212 m, on soil, 25 October 2021, LS Wang et al., 21-70536 (KUN-L 79977); JAPAN. Chugoku, Okayama, Japanese Archipielago, Honshu. Ohtahara, Wake-cho, Wake-gun, 34°48.75′ N 134°9.12′ E, 209 m, on humus, on soil, 19 March 2019, M. Sugimoto 591 (F C0678879); TAIWAN. Ilan, Taiping Shan National Forest Cuifong Lake circle Trail, 1840 m, 2 August 2023, J.P. Huang 175, 176. Nantou, Ren’ai Township, along Provincial Highway 14 (Renhe Road) near Kunyang checkpoint, 24°07′00″ N, 121°16′00″ E, 3090 m, on soil, 7 November 2019, J.P. Huang et al. 21502d (F C0678933), 21502f (F C0678934), 21502a (F C0678931). Taichung, Heping District, Wuling National Forest Recreation Area, Wuling Farm, along trail from Wuling Lodge to waterfall, 24°24′00″ N, 121°18′00″ E, 2100 m, on soil, 8 November 2019, J.P. Huang et al., 21508f (F C0678956), 21508c (F C0678953), 21508h (F C0678957), 21508d (F C0678955). Yilan, Nan’ao Township, Taipingshan National Forest Recreation Area, Chinese Hemlock Trail, 24°29′00″ N, 121°32′00″ E, 1900 m, on bark, 9 November 2019, J.P. Huang et al. 21513a (F C0678974). Yilan, Nan’ao Township, Taipingshan National Forest Recreation Area, Cueifong Lake Circular Trail, 24°30′55″ N, 121°36′21″ E, 1800–2000 m, on soil, 10 November 2019, J.P. Huang et al. 21518a (F C0678991), 21518c (F C0678990). Yilan, Nan’ao Township, Taipingshan National Forest Recreation Area, Taiwan Beech Trail, ca. 24°30′00″ N, 121°37′00″ E, 1800–1900 m, on soil, 10 November 2019, J.P. Huang et al. 21517b (F C0678987); THAILAND. Phitsanulok Province, Phu Hin Rong Kla National Park, Lan Hin Taek area, 16°59′13.0″ N, 100°59′50″ E, 1320 m, hill evergreen forest, on moss, 23 August 2023, K. Buaruang KBPH002 (RAMK 40319), KBPH005 (RAMK 40322), KBPH008 (RAMK 40325), W. Polyiam WPPH003 (RAMK 40320); V. Poengsungnoen VPPH004 (RAMK 40321), VPPH007 (RAMK 40324), on rock, W. Polyiam WPPH006 (RAMK 40323). Loei Province, Phu Luang Wildlife Sanctuary, Khok Nok Kraba Ranger Station, Tha Sala, Phu Ruea, 17°16′39.4″ N, 101°31′33.3″ E, 1555 m, lower montane scrub forest, on rock, 26 June 2008, W. Polyiam WPPL004 (RAMK 40341), WPPL008 (RAMK 40345), WPPL010 (RAMK 40347), K. Buaruang KBPL009 (RAMK 40346).

## 4. Discussion

### 4.1. Species Delimitation

The results support our hypotheses that RAD-seq data would reveal additional species within the *Cladia aggregata* complex and that geographic distance among continents limits gene flow between populations. Unlike previous studies, our analysis identified distinct Asian and American species within the *Cladia aggregata* complex, as evidenced by restricted gene flow between the two well-supported, reciprocally monophyletic groups. While earlier studies relied on the analysis of a limited number of molecular markers, the large volume of genomic data generated through next-generation sequencing in this study enabled more precise species delimitation within this group of closely related lichen-forming fungi. Most striking was the separation of most Asian specimens in a monophyletic clade, sister to *C. terebrata* and distinct from *C. aggregata* sensu stricto. Due to its clear delimitation, we propose that the Asia samples constitute a new species, designated as *Cladia asiatica*.

As the amount and resolution of data used for taxonomic inference have increased from morphology to single and multilocus phylogenies and now to genome-scale datasets, our understanding of species boundaries within the *Cladia aggregata* complex has become progressively more detailed. Early morphology-based studies, which also relied on chemical traits, treated the complex very broadly, recognizing it as only one, highly variable species [10] or delimiting eight species within the complex [11]. Subsequently, multi-locus molecular studies employing phylogenetic approaches expanded the diversity to 12 species, most of which were restricted to Australasia [19,20]. Only a few species included samples from outside the region: *C. cryptica* from Malaysia, *C. inflata* from Chile, and *C. gorgonea,* which occurs in both Australasia and La Réunion Island. The remaining Asian and American samples were still placed within *C. aggregata* [16]. Our study extends this work by combining a broader taxon sampling, particularly from China, Taiwan, and Japan, with a genome-scale dataset, providing the resolution necessary to accurately reconstruct evolutionary relationships within the *C. aggregata* complex across Asia and the Americas. The expanded dataset reveals limited gene flow between continents, likely due to geographic distance, and supports the notion that Asian and American populations represent distinct species.

Although some analyses suggest further structure between taxa from Thailand and the remaining Asian samples, or between South American taxa and Caribbean taxa, we refrain from formally delimiting additional species. The Thai samples form a distinct monophyletic group in some analyses but cluster within the broader Asian clade in others. They also exhibit partial separation in the PCA and in some fastSTRUCTURE K values, yet most analyses reveal admixture between Thailand and East Asia. Given this inconsistent signal and potential gene flow, we do not consider the Thai population to be a distinct species. Similarly, although South American and Caribbean samples form separate clades in some trees, the low sample size from the Caribbean and the high genetic similarity between the two regions argue against further species delimitation. Both fastSTRUCTURE and fineRADstructure indicate greater relatedness between these populations than between Asian and American samples, consistent with either incomplete lineage sorting or limited gene flow. Although these populations represent distinct genetic clusters, the level of divergence does not justify recognizing them as separate species.

Our results indicate that all samples from East Asia and Thailand belong to a species distinct from *Cladia aggregata* sensu stricto. Jamaican specimens analyzed in this study form a well-supported clade that we interpret as representing *C. aggregata* sensu stricto, consistent with the type specimen originally described by Olof Peter Swartz from Jamaica [17]. Any sample clustering with this lineage, or not sufficiently differentiated from it, should continue to be recognized as *C. aggregata.* In contrast, all Asian samples form a strongly supported monophyletic clade across multiple phylogenetic methods. While population genetics methods and statistical tests also confirm a high degree of genetic divergence between samples from Asia and those from the Caribbean, including the Jamaican samples, indicating limited or no gene flow.

### 4.2. Biogeography

In the Bayesian Binary MCMC tree, most ancestral nodes were assigned to Australasia, consistent with previous research on the genus *Cladia*, which suggests that the genus first evolved in Australia [44]. The common ancestor of the Asian populations is probably from East Asia. There is some uncertainty regarding the common ancestor of the Caribbean and South American populations, and we cannot definitively determine whether this common ancestor occupied South America, the Caribbean, Australasia, or a combination of these regions.

Gene flow between Australasia and the Southern tip of South America explains the connection of a sample from Argentina (C0090665F) with Australasian samples. A similar pattern was observed in a study of *Pseudocyphellaria glabra* using RAD-seq data, in which some samples from Chile and from New Zealand were interpreted as resulting from long-distance dispersal via migratory birds or wind currents carrying spores west to east [45]. An earlier study on the circumarctic lichen-forming fungus *Porpidia flavicunda* found that the disjunct populations shared haplotypes and lacked fixed nucleotide polymorphisms corresponding to their geographical locations, likely due to long-distance dispersal [46]. The odd placement of a single Taiwan sample (C0678932F) as sister to a New Zealand sample and apart from the larger Asian clade was further investigated using ITS barcoding and confirmed not to be an error. Therefore, further sampling is necessary to verify whether this sample represents a second species in Taiwan.

Further sampling is required across Australasia to comprehensively assess the diversity of the *Cladia aggregata* complex. Previous studies have documented considerable morphological and chemical variation within New Zealand and Australia, and our results suggest the presence of distinct populations and potentially distinct species within Australasia. However, a broader, more geographically balanced sampling is needed to draw definitive conclusions. RAD-seq of more evenly and extensively sampled Australasia populations could reveal the true biodiversity of *C. aggregata* in that region.

## 5. Conclusions

Our phylogenomic and population genetic analyses based on RAD-seq data clarified species boundaries within the *C. aggregata* species complex and revealed a previously unknown species from East Asia and Thailand, here described as *Cladia asiatica*. The genome-scale data clearly distinguished Asian from American samples, a separation not seen in earlier multilocus studies. Population genetics results show little to no evidence of admixture or co-ancestry, likely due to limited gene flow across the continents. Biogeographic patterns derived from this larger dataset align with previous findings for the genus *Cladia*, indicating a diversification event that predates the more recent dispersals to Asia and the Americas. Overall, this work highlights the importance of genome-scale data for accurate species delimitation and the identification of previously overlooked diversity within the *C. aggregata* complex.

## Figures and Tables

**Table 1:** Results of ipyrad Assembly and Analysis of RAD-seq Data.

Number of Filtered Loci	5432
Number of SNPs	30,841
Percentage of Missing Data	snps matrix size: 78.96% missing sites. sequence matrix size: 86.65% missing sites.
Average Number of Loci Per Sample	1483.505
Average Number of Raw Reads	7,120,563
Average Number of Mapped Reads	187,517.5
Average Error Estimate	0.001671

**Table 2:** Analysis of Molecular Variance (AMOVA) and Monte Carlo Test for Samples of *C. aggregata* from East Asia, Thailand, South America, and the Caribbean.

Variation Between Groups	544.9762	79.19%
Variation Within Groups	143.2486	20.81%
Total Variation	688.2249	100%
Phi-samples-total	0.7918578	
Monte-Carlo Test *p* Value	0.001	
Average Variance of Simulated Groups	170.36	
Observed Variance Between Actual Groups	544.98	

## Data Availability

The RAD sequence data used in this study were deposited in the NCBI Sequence Read Archive (SRA) through the accession number PRJNA1337906. Accession numbers for RADseq raw sequences are listed in Table S3. All the scripts that were used in this study can be found on GitHub at: https://github.com/jddeabreu/Cladia_aggregata (accessed on 20 November 2025).

## Supplementary Materials

The following supporting information can be downloaded at https://www.mdpi.com/article/10.3390/jof12020090/s1. Table S1: Sample Identified as other species in the *C. aggregata* species complex; Table S2: The main compounds of each chemotype in the dataset and the geographic distribution of the samples within those chemotypes; Table S3: Collection Data and herbaria accession numbers for all sequenced individuals; Figure S1: A phylogenetic, maximum likelihood tree made with RAxML; Figure S2: A coalescent-based tree made with SVDquartets in PAUP.
