# Supplementary material for: High-Throughput Sequencing Supports Strong Geographical Patterns in the Cladia aggregata Complex (Ascomycota, Lecanorales) and Identifies the Asian Clade as an Independent Species
Source: J Fungi (Basel). 2026 Jan 28;12(2):90. doi: 10.3390/jof12020090 (PMC12941837; doi:10.3390/jof12020090)
Supplement: Supplementary file 1 [file jof-12-00090-s001.zip › jof-4046743-supplementary.pdf]

Table S1: Morphological and DNA barcoding identification of *Cladia* samples. List of samples identified as species other than *C. aggregata* sensu stricto.

Country	Accession Number	Collection Number	Species	How was it Identified?	nuITS GenBank accession number	Collector
Argentina	C0090665F	12026h	*C. inflata*	Morphologically	N/A	R. Guderley, H. T. Lumbsch & G. Vobis
New Zealand	C1011086F	4627	*C. inflata*	Morphologically	N/A	D. J. Blanchon & H. Nessia
Australia	C1013645F	19975 a	*C. blanchonii*	DNA Barcoding (Parnmen et al. 2013)	GQ500909	H. T. Lumbsch
Australia	C0392872F	2211	*C. terebrata*	Morphologically	N/A	H. T. Lumbsch, T. J. Widhelm, F. Grewe
New Zealand	C1011208F	61709	*C. terebrata*	DNA Barcoding (Parnmen et al., 2013)	KC148370	A. Knight
New Zealand	C1011219F	61710	*C. neocaledonica*	Morphologically	N/A	A. Knight
Australia	C2035641F	20012i	Initial: *C. aggregata* s. lat. Later: *C. neocaledonica*	Initial: DNA Barcoding (Parnmen et al., 2013) Later: Morphologically	JN115311	H. T. Lumbsch, S. Parnmen, & T. J. Widhelm
Peru	N/A	19346b	*C. aggregata*	DNA Barcoding (Parnmen et al., 2013)	JN115251	H. T. Lumbsch
New Caledonia	C2035148F	7539	*C. neocaledonica*	DNA Barcoding (Parnmen et al., 2013)	KC689747	K. Papong & H. T. Lumbsch

Table S2: Chemotype compounds and information about the distribution of the samples within each chemotype.

Chemotype	Major Compounds	Distribution / Samples	Notes
**1**	Barbatic acid, 4-O-demethylbarbatic acid	Broadly distributed	Main chemotype in the dataset
**2**	Stictic acid complex	2 samples: one previously identified as *C. blanchonii* [20], one as *C. inflata*	Both retained in study
**3**	Fumarprotocetraric acid, succinprotocetraric acid, protocetraric acid	1 Australian sample (current outgroup)	Only present in outgroup
**4**	Barbatic acid, thamnolic acid, 4-O-demethylbarbatic acid	3 Bolivian samples	Unique to Bolivia
**5**	Barbatic acid, 4-O-demethylbarbatic acid, fumarprotocetraric acid	1 Bolivia sample, 1 New Caledonia sample (previously *C. neocaledonica* [20]), 1 Thailand sample (RAMK40318)	Thailand sample excluded from study
**6**	Atranorin	Both previous outgroup samples, both from Australia	Outgroup; excluded from analyses
**7**	Fumarprotocetraric acid	1 New Zealand outgroup sample, 1 New Zealand sample (C1011230F)	Both excluded from analyses

Table S3: Collection Data and accession numbers for all sequenced individuals.

Taxa	Country	collectors	Collection number	Herbarium Accession Number	Herbarium	NCBI SRA Accession Number
Cladia inflata	Argentina	R. Guderley, H. T. Lumbsch & G. Vobis	12026h	C0090665F	F	SRR360901 73
Cladia blanchonii	Australia	H. T. Lumbsch	19975 a	C1013645F	F	SRR360901 72

Cladia neocaledonica	Australia	H. T. Lumbsch, S. Parnmen, & T. J. Widhelm	20012i	C2035641F	F	SRR36090161
Cladia terebrata	Australia	H. T. Lumbsch, T. J. Widhelm, F. Grewe	2211	C0392872F	F	SRR36090150
Cladia ferdinandii	Australia	H. T. Lumbsch & J.P. Huang	20933a		F	SRR36090139
Cladia aggregata	Bolivia	Kukwa	9380	Ex UGDA L-18992	Ex UGDA L	SRR36090128
Cladia aggregata	Bolivia	Kukwa	9838	Ex UGDA L-19013	Ex UGDA L	SRR36090117
Cladia aggregata	Bolivia	Kukwa	9589	Ex UGDA L-19003	Ex UGDA L	SRR36090106
Cladia aggregata	Bolivia	Kukwa	10043	Ex UGDA L-19022	Ex UGDA L	SRR36090095
Cladia aggregata	Bolivia	Kukwa	9609	Ex UGDA L-19005	Ex UGDA L	SRR36090084
Cladia aggregata	Bolivia	Kukwa	8435	Ex UGDA L-18934	Ex UGDA L	SRR36090171
Cladia aggregata	Bolivia	Kukwa	16911	Ex UGDA L-62890	Ex UGDA L	SRR36090170
Cladia asiatica	China	LS Wang, XY Wang, X Ye, YY Zhang	15-46896	49783	KUN-L	SRR36090169
Cladia asiatica	China	LS Wang, XY Wang, LJ Li	19-63233	66707	KUN-L	SRR36090168
Cladia asiatica	China	LS Wang, XY Wang, AC Yin, LJ Li, LF Zeng	19-62809	66350	KUN-L	SRR36090167
Cladia asiatica	China	XY Wang, YY Zhang, CM Xie	21-69528	77570	KUN-L	SRR36090166
Cladia asiatica	China	XY Wang, AC Yin, CM Xie, M Ai	XY21-11	80438	KUN-L	SRR36090165

Cladia asiatica	China	XY Wang, M Ai	XY21-605	80139	KUN-L	SRR36090164
Cladia asiatica	China	XY Wang, M Ai	XY21-689	80223	KUN-L	SRR36090163
Cladia asiatica	China	LS Wang, XY Wang, Liu Dong, Shi Haixia, YY Zhang, MX Yang	14-43149	44955	KUN-L	SRR36090162
Cladia asiatica	China	D Liu	13-40004	20900	KUN-L	SRR36090160
Cladia asiatica	China	LS Wang, LJ Li, AC Yin	18-60455	63963	KUN-L	SRR36090159
Cladia asiatica	China	LS Wang, LJ Li, AC Yin	18-60722	64230	KUN-L	SRR36090158
Cladia asiatica	China	LS Wang, XY Wang, YY Zhang, AC Yin, LQ Jiang	18-59051	63385	KUN-L	SRR36090157
Cladia asiatica	China	LS Wang, XY Wang, Shi Haixia, YY Zhang, X Ye	14-43698	45566	KUN-L	SRR36090156
Cladia asiatica	China	XY Wang, AC Yin	18-61989	65524	KUN-L	SRR36090155
Cladia asiatica	China	LS Wang, D Liu, MX Yang, LT Wang, GH Zhao, LJ Li, ZJ Jiang	19-65746	70254	KUN-L	SRR36090154
Cladia asiatica	China	XY Wang, SY Wang	XY23-154	88761	KUN-L	SRR36090153
Cladia asiatica	China	XY Wang, AC Yin, CM Xie, M Ai	XY21-23	80450	KUN-L	SRR36090152
Cladia asiatica	China	LS Wang, XY Wang, LJ Li	19-63159	66633	KUN-L	SRR36090151
Cladia asiatica	China	LS Wang, XY Wang, YY Zhang, CM Xie	21-69695	77737	KUN-L	SRR36090149
Cladia asiatica	China	XY Wang, YY Zhang, CM Xie	21-69652	77694	KUN-L	SRR36090148

Cladia asiatica	China	X Ye, WC Wang	15-47927	50826	KUN-L	SRR36090147
Cladia asiatica	China	LS Wang, XY Wang	15-49383	52381	KUN-L	SRR36090146
Cladia asiatica	China	XY Wang, M Ai	XY22-1389	85207	KUN-L	SRR36090145
Cladia asiatica	China	LS Wang, CM Xie, Q Wang	21-70633	80074	KUN-L	SRR36090144
Cladia asiatica	China	LS Wang, CM Xie, Q Wang	21-70536	79977	KUN-L	SRR36090143
Cladia asiatica	China	XY Wang, YY Zhang, CM Xie	21-69543	77585	KUN-L	SRR36090142
Cladia asiatica	China	LS Wang, XY Wang, LJ Li	19-63209	66683	KUN-L	SRR36090141
Cladia aggregata	Colombia	Coca	11912			SRR36090140
Cladia aggregata	Colombia	S. Gómez-Gómez	268			SRR36090138
Cladia aggregata	Colombia	F. Fajardo	2007A			SRR36090137
Cladia aggregata	Cuba	Mercado-Diaz	2729a		UPR	SRR36090136
Cladia aggregata	Cuba	Mercado-Diaz	2843		UPR	SRR36090135
Cladia aggregata	Ecuador	Nelsen & White	7078		F	SRR36090134
Cladia aggregata	Jamaica	Mercado-Diaz	3395		UPR	SRR36090133
Cladia aggregata	Jamaica	Mercado-Diaz	3456a		UPR	SRR36090132

Cladia aggregata	Jamaica	Mercado-Diaz	3490		UPR	SRR36090131
Cladia aggregata	Jamaica	Mercado-Diaz	3414a		UPR	SRR36090130
Cladia aggregata	Jamaica	Mercado-Diaz	3397a		UPR	SRR36090129
Cladia asiatica	Japan	M. Sugimoto	591	C0678879F	F	SRR36090127
Cladia neocaledonica	New Caledonia	K. Papong & H. T. Lumbsch	7539	C2035148F	F	SRR36090126
Cladia aggregata	New Caledonia	K. Papong & H. T. Lumbsch	7504	C2035477F	F	SRR36090125
Cladia aggregata	New Caledonia	K. Papong & H. T. Lumbsch	7537k	C2035478F	F	SRR36090124
Cladia terebrata	New Zealand	A. Knight	61709	C1011208F	F	SRR36090123
Cladia aggregata	New Zealand	A. Knight	61707	C1011230F	F	SRR36090122
Cladia neocaledonica	New Zealand	A. Knight	61710	C1011219F	F	SRR36090121
Cladia inflata	New Zealand	D. J. Blanchon & H. Nessia	4627	C1011086F	F	SRR36090120
Cladia aggregata	New Zealand	P. J. de Lange	PLD 14130	C0370945F	F	SRR36090119
Cladia aggregata	New Zealand	P. J. de Lange & J. R. Rolfe	12705	C0346857F	F	SRR36090118
Cladia aggregata	New Zealand	P. J. de Lange	PDL 13154	C0370941F	F	SRR36090116
Cladia aggregata	Peru	Lumbsch	19346b		F	SRR36090115

Cladia aggregata	Puerto Rico	Mercado-Diaz	3641		UPR	SRR36090114
Cladia aggregata	Puerto Rico	Mercado-Diaz	2363		UPR	SRR36090113
Cladia asiatica	Taiwan	J.P. Huang et al.	21513a	C0678974F	F	SRR36090112
Cladia asiatica	Taiwan	J.P. Huang et al.	21502d	C0678933F	F	SRR36090111
Cladia aggregata	Taiwan	J.P. Huang et al.	21502b	C0678932F	F	SRR36090110
Cladia asiatica	Taiwan	J.P. Huang et al.	21508f	C0678956F	F	SRR36090109
Cladia asiatica	Taiwan	J.P. Huang et al.	21517b	C0678987F	F	SRR36090108
Cladia asiatica	Taiwan	J.P. Huang et al.	21518a	C0678991F	F	SRR36090107
Cladia asiatica	Taiwan	J.P. Huang et al.	21502f	C0678934F	F	SRR36090105
Cladia asiatica	Taiwan	J.P. Huang	175			SRR36090104
Cladia asiatica	Taiwan	J.P. Huang	176			SRR36090103
Cladia asiatica	Taiwan	J.P. Huang et al.	21508c	C0678953F	F	SRR36090102
Cladia asiatica	Taiwan	J.P. Huang et al.	21518c	C0678990F	F	SRR36090101
Cladia asiatica	Taiwan	J.P. Huang et al.	21508h	C0678957F	F	SRR36090100
Cladia asiatica	Taiwan	J.P. Huang et al.	21508d	C0678955F	F	SRR36090099

Cladia asiatica	Taiwan	J.P. Huang et al.	21502a	C0678931F	F	SRR36090098
Pulchrocladia retipora	Australia	H. T. Lumbsch, T. J. Widhelm, F. Grewe	2150	C0392859F	F	SRR36090097
Cladia asiatica	Thailand	Vasun Poengsungnoen	VPPH001	RAMK40318	RAMK	SRR36090096
Cladia asiatica	Thailand	Kawinnat Buaruang	KBPH002	RAMK40319	RAMK	SRR36090094
Cladia asiatica	Thailand	Wetchasart Polyiam	WPPH003	RAMK40320	RAMK	SRR36090093
Cladia asiatica	Thailand	Vasun Poengsungnoen	VPPH004	RAMK40321	RAMK	SRR36090092
Cladia asiatica	Thailand	Kawinnat Buaruang	KBPH005	RAMK40322	RAMK	SRR36090091
Cladia asiatica	Thailand	Vasun Poengsungnoen	VPPH007	RAMK40324	RAMK	SRR36090090
Cladia asiatica	Thailand	Kawinnat Buaruang	KBPH008	RAMK40325	RAMK	SRR36090089
Cladia asiatica	Thailand	Wetchasart Polyiam	WPPH006	RAMK40323	RAMK	SRR36090088
Cladia asiatica	Thailand	Kawinnat Buaruang	KBPL001	RAMK40338	RAMK	SRR36090087
Cladia asiatica	Thailand	Kawinnat Buaruang	KBPL005	RAMK40342	RAMK	SRR36090086
Cladia asiatica	Thailand	Kawinnat Buaruang	KBPL007	RAMK40343	RAMK	SRR36090085
Cladia asiatica	Thailand	Wetchasart Polyiam	WPPL006	RAMK40344	RAMK	SRR36090083
Cladia asiatica	Thailand	Wetchasart Polyiam	WPPL004	RAMK40341	RAMK	SRR36090082

Cladia asiatica	Thailand	Wetchasart Polyiam	WPPL008	RAMK40345	RAMK	SRR36090081
Cladia asiatica	Thailand	Kawinnat Buaruang	KBPL009	RAMK40346	RAMK	SRR36090080
Cladia asiatica	Thailand	Wetchasart Polyiam	WPPL010	RAMK40347	RAMK	SRR36090079

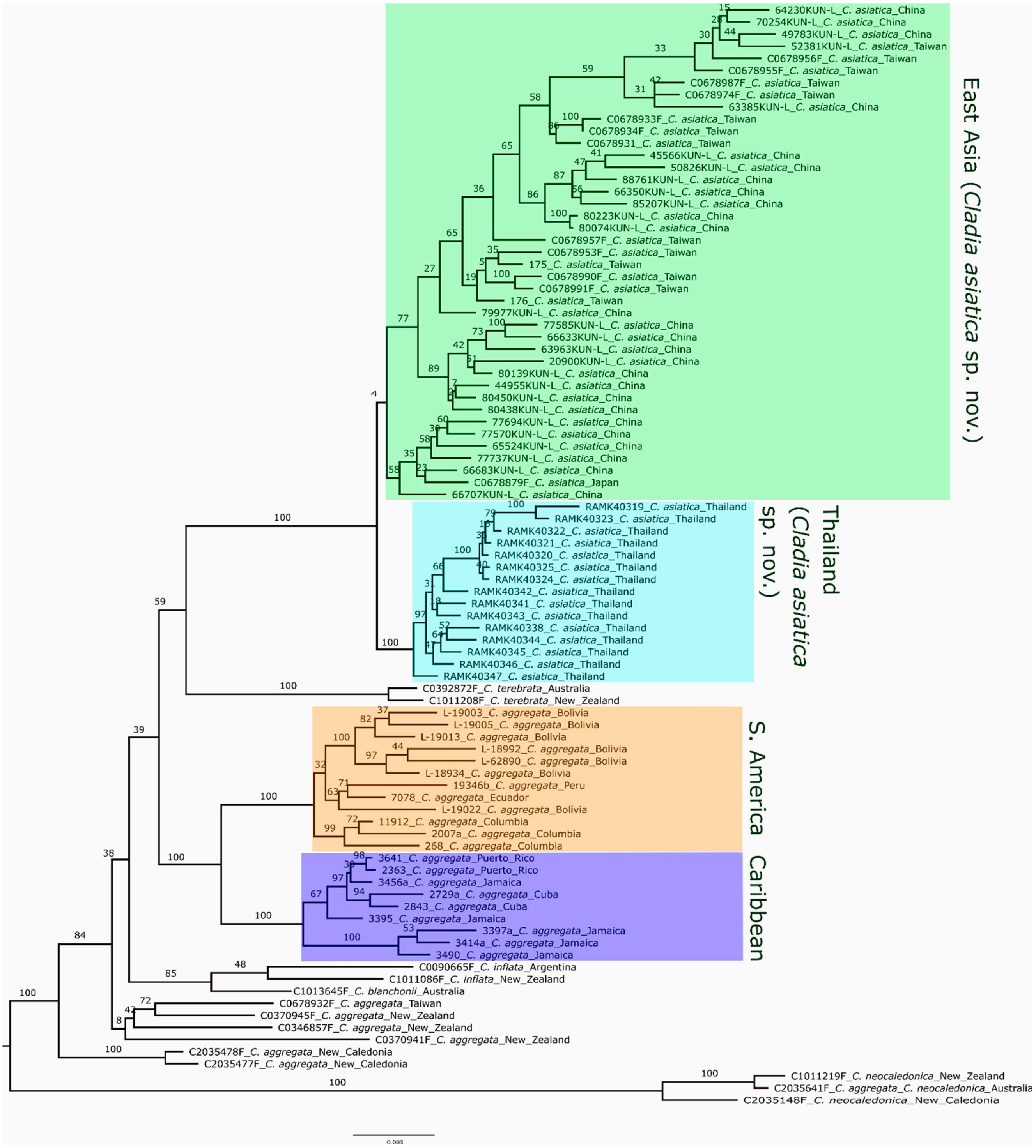

Figure S1: A maximum likelihood tree calculated with the reduced taxon set of 91 samples using RAxML. The tree is displayed as a phylogram, with C. neocaledonica forming an outgroup clade. Bootstrap values above 75 are located near the nodes, and branch lengths represent the number of substitutions per site, as indicated by the scale. Each sample ID is represented in the following format: the accession number, if available, or collection number, followed by the species name, and the country where the sample was collected. The Asian clade corresponds to the here-described Cladia asiatica sp. nov.

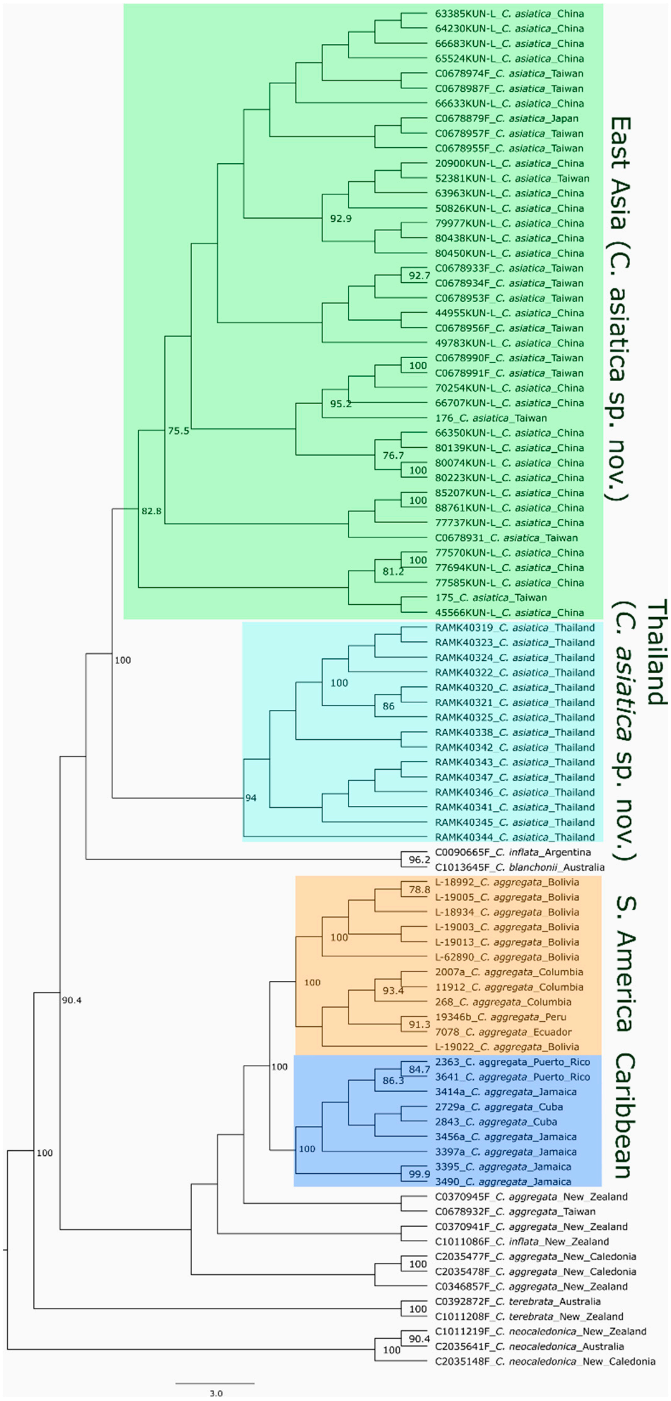

Figure S2: A coalescent-based tree calculated with SVDquartets in PAUP. The tree is displayed as a cladogram, with C. neocaledonica forming an outgroup clade. Bootstrap values above 75 are located near the nodes. Bootstrap values were assigned using CASTER-site with a phylip file as input data. The geographic regions of well-supported clades are highlighted. Each sample ID is represented in the following format: the accession number, if available, or collection number, followed by the species name, and the country where the sample was collected. The Asian clade corresponds to the here-described Cladia asiatica sp. nov.
